# Characteristics of homologous recombination repair pathway genes mutation in ovarian cancers

**DOI:** 10.1002/cai2.27

**Published:** 2022-09-20

**Authors:** Zongbi Yi, Min Chen, Shaoxing Sun, Chunxu Yang, Zijie Mei, Hui Yang, Qingming Xiang, Hui Qiu

**Affiliations:** ^1^ Department of Radiation and Medical Oncology, Hubei Key Laboratory of Tumour Biological Behaviors, Hubei Cancer Clinical Study Center Zhongnan Hospital of Wuhan University Wuhan China

**Keywords:** ovarian cancer, genetic testing, somatic mutation, homologous recombination repair, platinum‐based therapy

## Abstract

**Background:**

Few studies have investigated the characteristics of non‐*BRCA* homologous recombination repair (HRR) pathway somatic mutations, and the impact of these mutations on efficacy of treatment in ovarian cancer patients is not clear. Therefore, we conducted this study to analyze the frequency and spectrum of somatic mutations in HRR pathway genes in patients with ovarian cancer and to examine the relationships between somatic mutations in HRR pathway genes and their effects on the efficacy of platinum‐based chemotherapy.

**Methods:**

We performed targeted sequencing of 688 genes related to the occurrence, development, treatment, and prognosis of solid tumors. Somatic mutations were identified by paired analysis of tumor tissue and germline DNA in blood cells.

**Results:**

A total of 38 patients with ovarian cancer were included in the study, and 35 (92.1%) patients were diagnosed with high‐grade serous carcinoma. All patients exhibited somatic mutations in the tumor tissue samples. The commonly mutated genes were *TP53* (73.7%), *BRCA2* (55.3%), *NF1* (52.6%), *BRCA1* (47.4%), and *CDH1* (47.4%). Overall, 71.1% of the patients exhibited mutation in at least one HRR pathway gene. The most frequently altered HRR genes were *BRCA2* (55.3%), followed by *BRCA1* (47.4%), *ATM* (44.7%), *BARD1* (42.1%), and *CHEK1* (36.8%). The median progression‐free survival (PFS) in patients with HRR pathway mutation was 36.0 months compared with 13.6 months in patients with no HRR pathway mutation (hazard ratio [HR], 0.25; 95% confidence interval [CI], 0.08–0.77; *p* = 0.016). Patients harboring *BRCA1/2* and/or *CDK12* mutations displayed a longer PFS (median, 36.0 months) compared with patients with no *BRCA1/2* or *CDK12* mutation (median, 13.6 months; HR, 0.21; 95% CI, 0.07–0.61; *p* = 0.004). In multivariate analysis Cox proportional hazards models, after adjustment for tumor stage at diagnosis and histology of initial diagnosis, patients with HRR pathway mutation had a longer PFS than patients with HRR wild‐type genes (*p* = 0.006).

**Conclusions:**

HRR pathway somatic mutations are common in Chinese patients with ovarian cancer. HRR pathway somatic mutations were associated with improved sensitivity to platinum‐based chemotherapy. Large‐scale prospective studies are needed to verify our findings.

## Graphical abstract

This study analyzed the spectrum of somatic mutations in homologous recombination repair (HRR) pathway genes in patients with ovarian cancer and examined the relationships between somatic mutations in HRR pathway genes and their effects on the efficacy of platinum‐based chemotherapy. The results revealed that HRR pathway somatic mutations are common in Chinese patients with ovarian cancer and associated with improved sensitivity to platinum‐based chemotherapy.

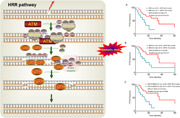

**Abbreviations**
CI: confidence interval
FIGO: The International Federation of Gynecology and Obstetrics
HGSC: high‐grade serous carcinoma
HR: hazard ratio
HRR: homologous recombination repair
PARP: poly adenosine diphosphate‐ribose polymerase
PFS: progression‐free survival

## 1 BACKGROUND

Ovarian cancer is the third most common gynecologic malignant tumor in the world, and its mortality ranks first among gynecological tumors [1]. The incidence and mortality rates of ovarian cancer in China are also increasing year by year. Ovarian cancer patients have no typical early clinical symptoms [2]. Additionally, there is a lack of effective screening and early diagnosis methods for ovarian cancer patients. As a result, more than 80% cases of ovarian cancer patients are diagnosed at an advanced stage with distant metastasis [2, 3]. This substantially reduces the cure rate of ovarian cancer, and the 5‐year survival rate is less than 30% [1, 3].

The current treatment methods for patients with ovarian cancer mainly include surgery, chemotherapy, radiotherapy, and molecular targeted therapy [1]. The standard treatment for ovarian cancer patients is surgery combined with postoperative platinum‐based chemotherapy [1]. Research on the molecular mechanism of ovarian cancer has led to the identification of poly adenosine diphosphate‐ribose polymerase (PARP) inhibitors as an important targeted therapy for ovarian cancer patients, but these inhibitors are mainly used in patients with homologous recombination deficiency or patients with platinum‐sensitive recurrent tumors [1, 4]. Other therapies, such as immunotherapy and antiangiogenesis therapy, have limited efficacy in patients with ovarian cancer [1, 5]. Therefore, finding new therapeutic targets or combined treatment strategies is very important to improve the survival of patients with recurrent and metastatic ovarian cancer.

Homologous recombination repair (HRR) pathway gene mutations are found in approximately 30% of ovarian carcinoma patients [6, 7]. *BRCA1* and *BRCA2* (*BRCA1/2*) are the most common HRR gene mutations [8, 9, 10]. Ovarian cancer patients with *BRCA1* or *BRCA2* mutations have a better response to platinum chemotherapy and longer progression‐free survival (PFS) [6]. Few studies have examined the characteristics of somatic mutations in HRR pathway genes other than *BRCA1/2*, and the impact of these mutations on treatment efficacy and prognosis in patients with ovarian cancer is not clear. Therefore, we conducted this study to analyze the frequency and spectrum of somatic mutations in HRR pathway genes in patients with advanced ovarian cancer and to examine the relationships between mutations in HRR pathway genes and their effects on the efficacy of platinum‐based chemotherapy.

## 2 MATERIALS AND METHODS

### 2.1 Patients

This study included tumor tissues from patients with ovarian cancer treated at the Department of Radiation and Medical Oncology, Zhongnan Hospital of Wuhan University between March 2017 and October 2019. The inclusion criteria were as follows: (1) patients who were at least 18 years old; (2) patients who had a histologic/cytologic diagnosis of invasive ovarian cancer; and (3) patients who had complete clinicopathological data. The exclusion criteria were as follows: (1) patients who had a histologic/cytologic diagnosis of sarcoma ovarian cancer; and (2) patients with insufficient tumor tissue for DNA sequencing. This study was approved by the Ethics Committee of Zhongnan Hospital of Wuhan University. All patients provided written informed consent.

### 2.2 Sample collection and next‐generation sequencing

Gene testing for ovarian cancer patients was ordered by the treating physician to identify clinically relevant mutations that could potentially aid in treatment decisions. Tumor tissue samples were collected at the time of recurrence and metastasis was identified. We performed targeted sequencing of 688 genes related to the occurrence, development, treatment, and prognosis of solid tumors. Somatic mutations were identified by paired analysis of tumor tissue and germline DNA in blood cells. The MGISEQ‐2000RS High Output kit (BGI) and MGISEQ‐2000 (BGI) were used for DNA sequencing. DNA library preparation, hybrid capture, sequencing, and analysis were performed as previously described [11].

### 2.3 Statistical analysis

Primary platinum sensitivity was defined as a complete response during adjuvant chemotherapy and clinical remission for at least 6 months after completion of chemotherapy. Primary platinum resistance was defined as progressive disease on platinum therapy, less than a complete response to platinum therapy or progression within 6 months of completing platinum therapy. PFS was defined as the time from the completion of platinum‐based chemotherapy to the date of disease progression or death of any cause. Patients without an endpoint (progression or death events) were censored at the date of the last follow‐up. All patients were regularly followed‐up every 3 months within 2 years and every 6 months 2‐5 years after the surgery. The efficacy, safety, and survival data of advanced ovarian cancer patients were followed‐up every 2‐3 months. The last follow‐up time was June 15, 2022. Kaplan–Meier survival plots were generated using HRR pathway mutation status, and curves were compared using log‐rank tests. A Cox proportional hazards model was used to analyze the association between PFS and HRR pathway mutations and clinical characteristics. All statistical tests were two‐sided, and *p* values less than 0.05 were considered significant. Statistical analyses were performed using SPSS version 23.0 (IBM Corporation) or GraphPad Prism 7.0 (GraphPad Software).

## 3 RESULTS

### 3.1 Patient characteristics

A total of 38 patients with ovarian cancer were included in the study. Patient characteristics are summarized in Table 1. There were 35 patients (92.1%) diagnosed with high‐grade serous carcinoma (HGSC), 2 patients (5.3%) diagnosed with clear cell carcinoma, and 1 patient (2.6%) diagnosed with ovarian endometrioid carcinoma. The median patient age at initial diagnosis was 56 years (range: 30–76 years).

**Table 1:** Patient characteristics (*n* = 38)

Characteristics	No.	Percentage (%)
**Age, years**		
<40	1	2.6
40–49	8	21.1
50–59	18	47.4
≥60 years	11	28.9
**Family history of breast or ovarian cancers**		
Yes	1	2.6
No	34	89.5
Unknown	3	7.9
**FIGO stage**		
I	7	18.4
II	2	5.3
III	22	57.9
IV	7	18.4
**Received the ovarian cytoreductive surgery**		
Yes	37	97.4
No	1	2.6
**Histological type**		
High‐grade serous carcinoma	35	92.1
Clear cell	2	5.3
Other	1	2.6
**HRR gene mutations**		
Yes	28	73.7
No	10	26.3
**Platinum response**		
Platinum sensitive	33	86.8
Platinum resistant	5	13.2

Abbreviations: FIGO, The International Federation of Gynecology and Obstetrics. HRR, homologous recombination repair.

### 3.2 Genomic characterization and HRR mutation rate

**Figure 1:**
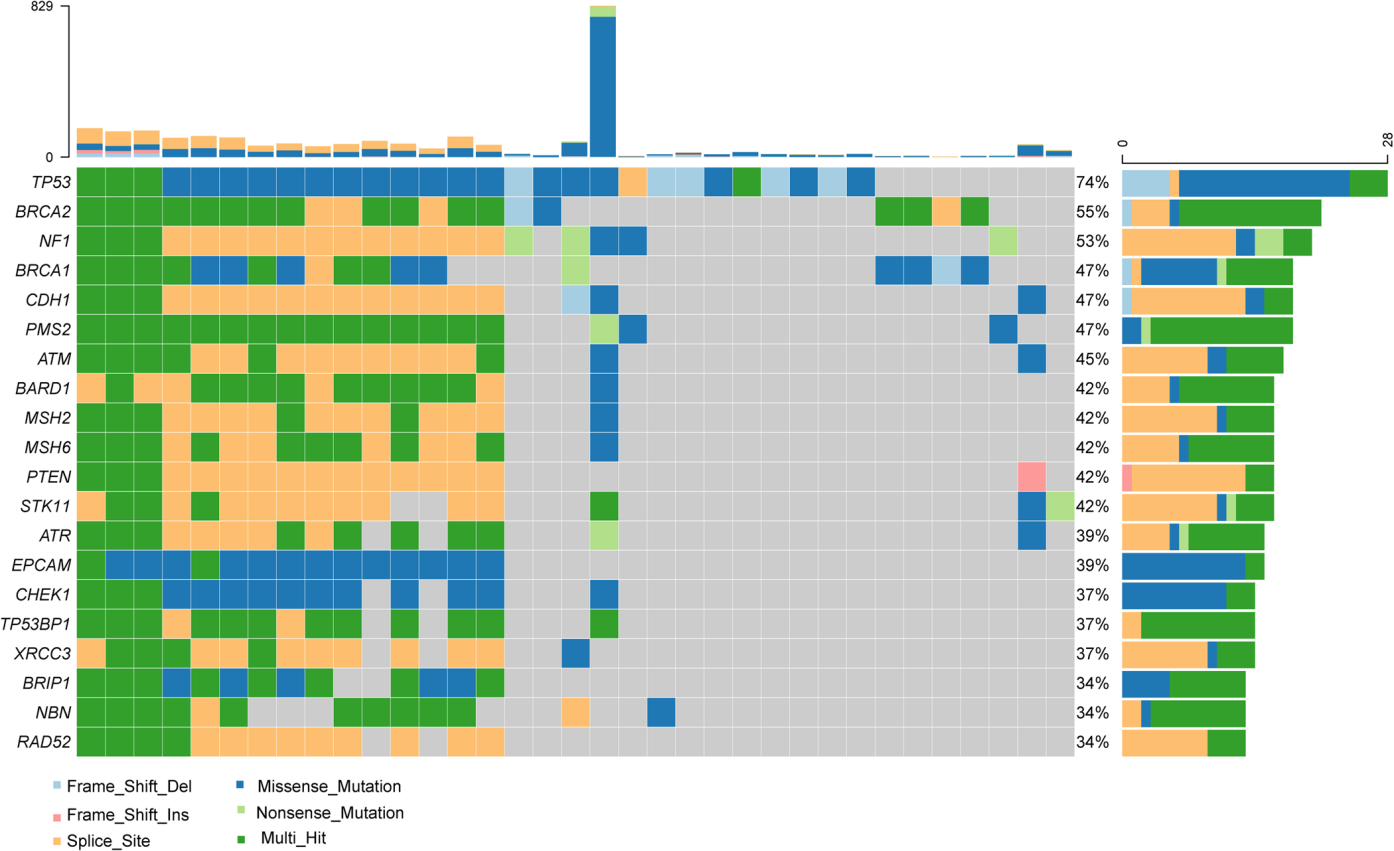
Mutation spectrum of the top 20 genes

We performed target region capture‐based next‐generation sequencing to evaluate the status of somatic mutations. All patients (100%) showed somatic mutations in the tumor tissue samples. The commonly mutated genes were *TP53, BRCA2, NF1, BRCA1* and *CDH1*, and mutations in these genes were detected in 28 (73.7%), 21 (55.3%), 20 (52.6%), 18 (47.4%), and 18 (47.4%) patients, respectively (Figure 1).

**Figure 2:**
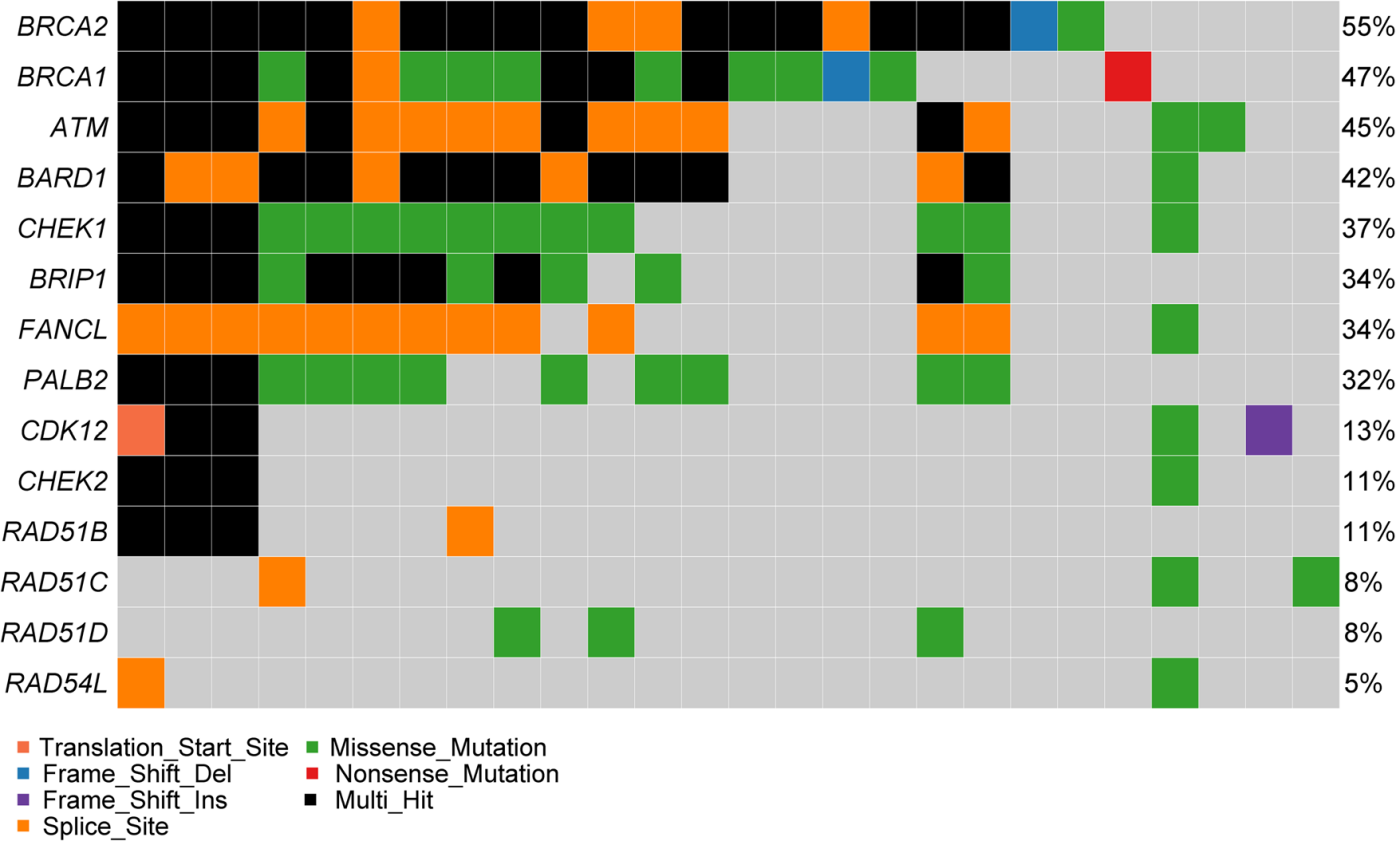
Mutation spectrum of the 14 homologous recombination DNA repair pathway genes

**Figure 3:**
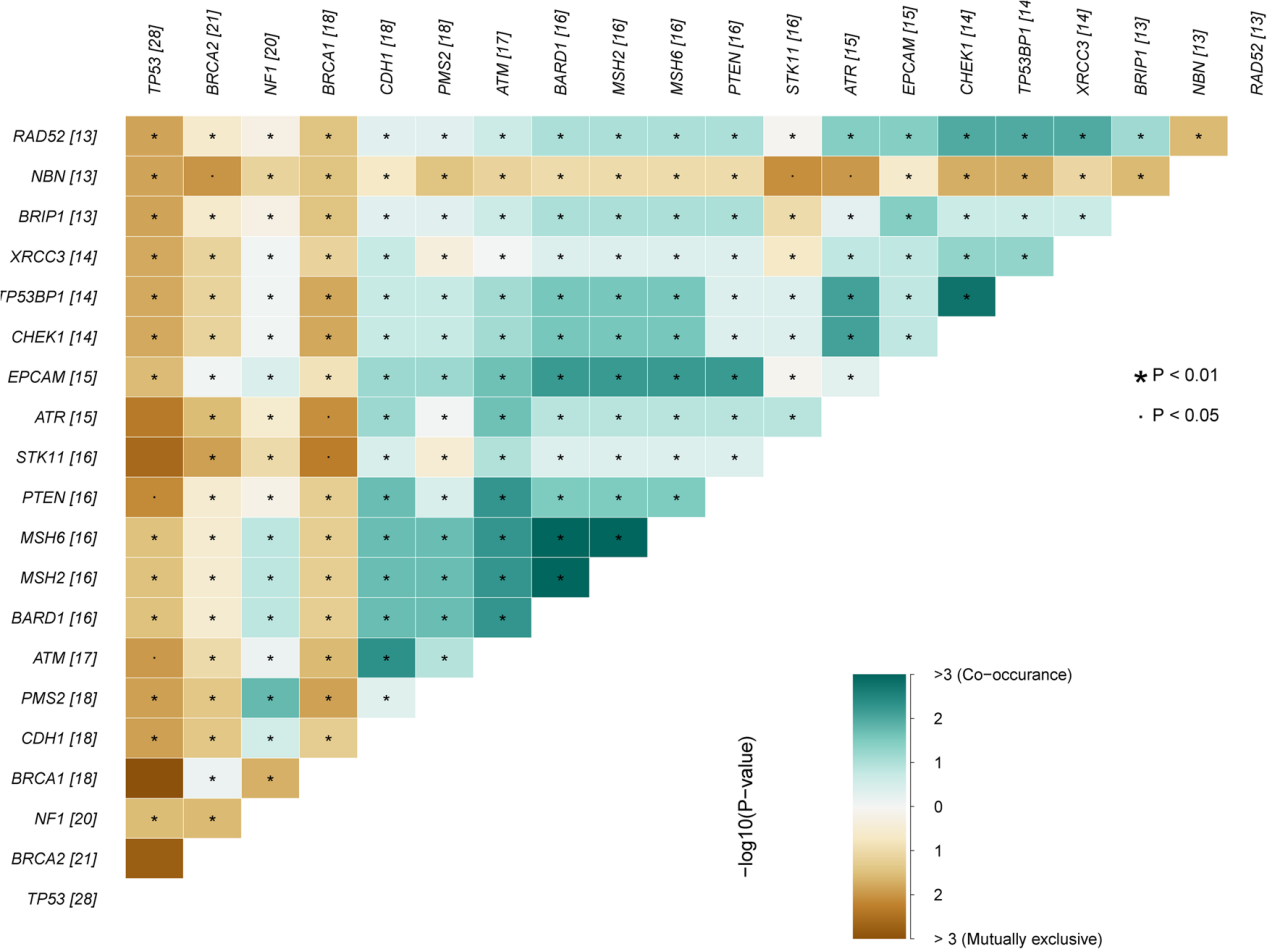
Somatic interactions of the top 20 genes. Pairwise Fisher's exact test was used to detect significant gene pairs.

Overall, 27 (71.1%) of the 38 enrolled patients exhibited mutations in at least 1 of the following 14 HRR pathway genes: *ATM*, *BRCA1*, *BRCA2*, *BARD1*, *BRIP1*, *CDK12*, *CHEK1*, *CHEK2*, *FANCL*, *PALB2*, *RAD51B*, *RAD51C*, *RAD51D*, and *RAD54L*. The most frequently altered HRR gene was *BRCA2* (55.3%), followed by *BRCA1* (47.4%), *ATM* (44.7%), *BARD1* (42.1%), and *CHEK1* (36.8%). The genomic alterations identified in HRR pathway genes in the 38 patients are shown in Figure 2. *BRCA1/2* mutations tended to be exclusive with other mutations. The most frequent mutually exclusive mutated genes with *BRCA2* were *BRCA1*, *CDH1*, *PMS2*, *ATM*, *BARD1*, *MSH2/6*, and *PTEN*. In contrast, *ATM* mutations co‐occurred with mutations in *BARD1*, *MSH2*, *MSH6*, *PTEN*, *STK11*, and other genes (Figure 3).

*CDK12* is an important HRR pathway factor that is related to resistance to chemotherapy and PARP inhibitors, but *CDK12* gene mutation is related to sensitivity to immunotherapy. We analyzed the frequency and spectrum of *CDK12* mutations. We found that five (13.2%) patients harbored at least one *CDK12* somatic mutation. The distribution of mutations was nonuniform across the gene.

### 3.3 Responses to platinum‐based chemotherapy by mutation status of HRR pathway genes

All 38 ovarian cancer patients received platinum‐based chemotherapy. In total, 33 (86.8%) patients were sensitive to platinum‐based chemotherapy and 5 (13.2%) patients were resistant to platinum‐based chemotherapy.

**Figure 4:**
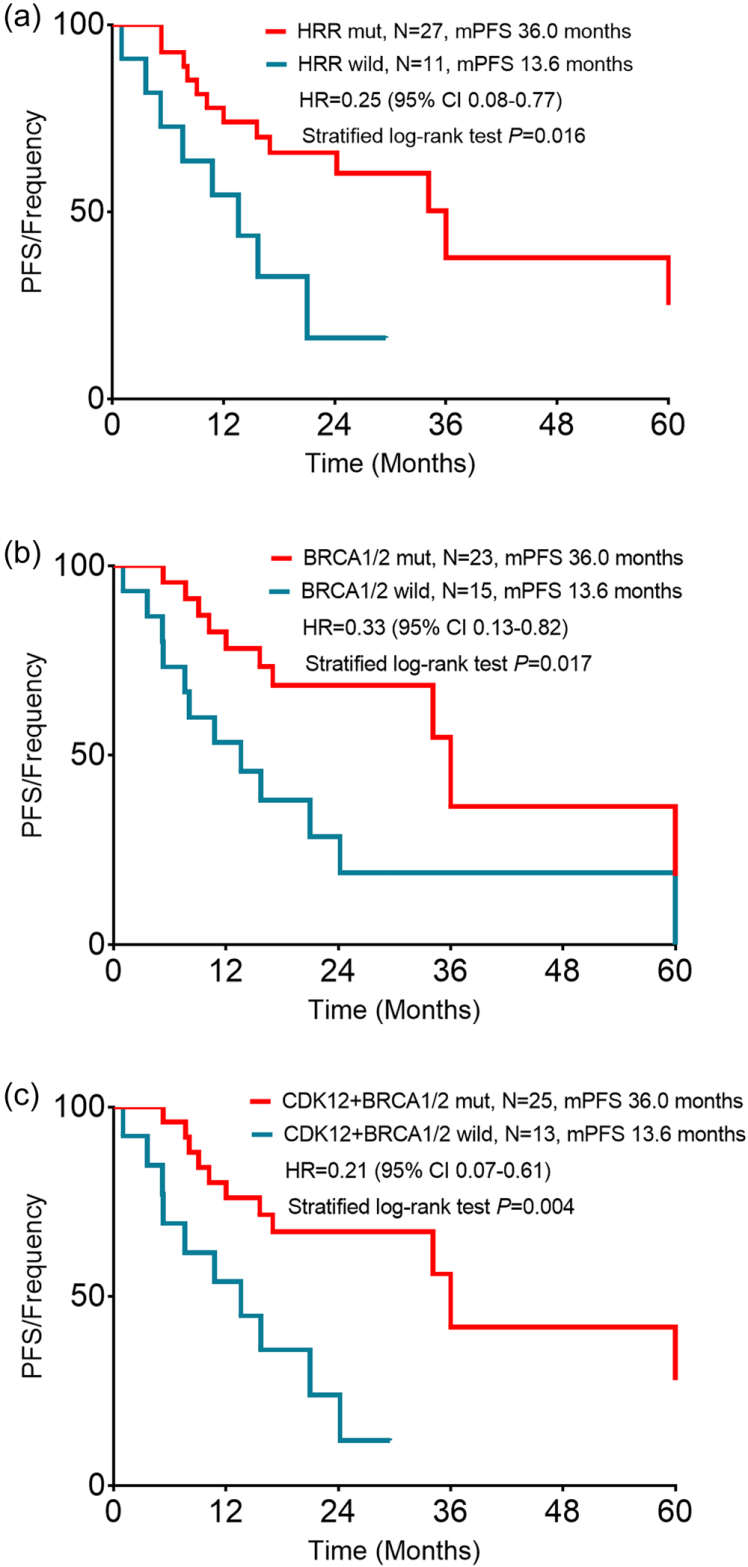
Impact of HRR mutation on the response to platinum‐based therapy. (a) PFS of patients who underwent platinum‐based treatment based on the presence of HRR mutation. (b) PFS of patients who underwent platinum‐based treatment based on the presence of *BRCA1/2* mutation. (c) PFS of patients who underwent platinum‐based treatment based on the presence of *CDK12* + *BRCA1/2* mutation. CI, confidence interval; HR, hazard ratio; HRR, homologous recombination repair; PFS, progression‐free survival.

The median follow‐up time was 27.8 months (range 1‐156 months). The median PFS in the patients with HRR pathway mutation was 36.0 months compared with 13.6 months in the patients with no HRR pathway mutation (hazard ratio [HR], 0.25; 95% confidence interval [CI], 0.08–0.77; *p* = 0.016; Figure 4a). The patients harboring *BRCA1/2* mutations (median, 36.0 months) displayed a longer PFS than those with wild‐type *BRCA1/2* (median, 13.6 months; HR, 0.33; 95% CI, 0.13–0.82; *p* = 0.017; Figure 4b). We further analyzed the association between *CDK12* and PFS. The patients harboring *BRCA1/2* and/or *CDK12* mutation displayed a longer PFS (median, 36.0 months) compared with patients with no *BRCA1/2* or *CDK12* mutation (the median PFS of 13.6 months; HR, 0.21; 95% CI, 0.07–0.61; *p* = 0.004; Figure 4c).

The results of the univariate and multivariate Cox regression analysis with clinical characteristics and HRR mutation status are shown in Table 2. In multivariate analysis Cox proportional hazards models, after adjustment for tumor stage at diagnosis and histology of initial diagnosis, patients with HRR pathway mutation showed a longer PFS than patients with HRR wild‐type genes (*p* = 0.006). Patients with *BRCA1/2* mutation had a longer PFS than patients with *BRCA1/2* wild‐type (*p* = 0.001). Patients with *BRCA1/2* and/or *CDK12* mutation had a longer PFS than patients with *BRCA1/2* and *CDK12* wild‐type (*p* = 0.001).

**Table 2:** Cox regression analysis of the associations between the PFS and clinical characteristics and HRR gene status

Variable	Univariate analysis		Multivariable analysis^a^	
	HR (95% CI)	*p*	HR (95% CI)	*p*
HRR mutation status	0.33 (0.13–1.85)	0.022	0.23 (0.08–0.65)	0.006
*CDK12* and *BRCA1/2* mutation status	0.28 (0.11–0.71)	0.007	0.17 (0.06–0.48)	0.001
*BRCA1/2* mutation status	0.37 (0.16–0.86)	0.021	0.18 (0.06–0.49)	0.001
HRR mutations in non *BRCA1/2*	0.47 (0.19–1.13)	0.089	0.46 (0.18–1.15)	0.096

Abbreviations: PFS, progression‐free survival. HRR: homologous recombination DNA repair; HR: hazard ratio.

^a^ After adjustment for tumor stage at diagnose (stage 1‐2 versus stage 3‐4) and histology of initial diagnosis (high grade serous carcinoma versus others).

## 4 DISCUSSION

Ovarian cancer is a tumor that shows high heterogeneity [12]. Detection of genomic alterations of ovarian cancer patients is very important for personalized treatment. Here, we explored the spectrum of somatic mutations in 38 ovarian cancer patients. Most patients (92.1%) were diagnosed with HGSC ovarian cancer. Consistent with data from previous studies [13], *TP53* was the most frequently mutated gene and *TP53* mutations were detected in 73.7% patients in the present study. *TP53* is a well‐known tumor suppressor gene that is frequently mutated in various solid tumors [14]. *TP53* somatic mutation is detected in approximately 40%–60% of advanced serous ovarian cancers [9], which was lower than the rate in the present study [10]. The differences in the enrolled populations may explain these differences. A possible explanation for this discrepancy is that most of the patients enrolled in the present study had metastatic ovarian cancer with a high tumor burden and had received multiple anticancer therapies. Most somatic mutations in patients with advanced or metastatic ovarian cancer may be mutations acquired during drug treatment. In addition to *TP53* mutations, *BRCA2* (55.3%), *NF1* (52.6%), *BRCA1* (47.4%), and *CDH1* (47.4%) mutations were also commonly detected in ovarian cancer in the present study.

We identified HRR pathway somatic mutations in 71.1% patients in this study. The majority of HRR mutations occurred in *BRCA2* (55.3%), *BRCA1* (47.4%), *ATM* (44.7%), *BARD1* (42.1%), and *CHEK1* (36.8%). The results of the landscape of the mutations in HRR pathway were consistent with previous reports. However, the rate of HRR pathway somatic mutations was higher than previously reported rates [7, 9, 13, 15, 16, 17]. We identified mutations in every HRR gene in the panel, and the mutation rate would likely be higher if more genes in the HRR pathway were queried. Most somatic mutations in HRR pathway genes were in *BRCA1* or *BRCA2*. *BRCA1/2* mutations tend to be exclusive with other HRR pathway gene mutations, such as *CDH1*, *PMS2*, *ATM*, *BARD1*, *MSH2/6*, and *PTEN*. This may be because *BRCA1/2* mutation is a driving factor of ovarian cancer.

*CDK12* encodes a transcription‐related kinase that is involved in a variety of biological processes, including DNA damage repair and precursor mRNA splicing [18, 19]. *CDK12* deletion does not change overall transcription and it only affects some genes related to the DNA damage response [20, 21]. The most altered genes are those with a large number of exons, including *BRCA1*, *ATR*, *FANCI*, and *FANCD2*. Deletion of *CDK12* resulted in decreased *BRCA1* transcription and increased sensitivity to DNA damaging agents [22, 23]. The deletion rate of *CDK12* in patients with metastatic prostate cancer is approximately 3%–7% [18]. The inactivation of *CDK12* in prostate cancer is related to the resistance to endocrine therapy, paclitaxel, and PARP inhibitors and sensitivity to immunotherapy [24]. However, few studies have examined *CDK12* in patients with recurrent and metastatic ovarian cancer, and the somatic mutation characteristics of *CDK12* are not clear. We found that 13.2% patients harbored at least one *CDK12* somatic mutation. The distribution of mutations was nonuniform across the gene. Patients with *CDK12* and/or *BRCA1/2* mutation were more sensitive to platinum‐based chemotherapy.

Several clinical studies [25, 26, 27, 28, 29] have confirmed that patients with HRR pathway mutations are sensitive to PARP inhibitors. However, all of these studies focused on germline mutations. Few studies have reported the relationship between HRR pathway somatic mutations and the efficacy of platinum‐based chemotherapy or PARP inhibitors. We hypothesize that individuals with somatic mutations in HRR pathway will also have increased response rates to platinum‐based chemotherapy. Our results confirmed that somatic *BRCA1/2* mutations and other HRR pathway gene mutations were associated with improved platinum‐based chemotherapy sensitivity. Some limitations of this study must be acknowledged. First, FFS was retrospectively calculated, which may influence the survival analysis, as HRR somatic mutation status may change after distant metastasis. Second, the retrospective design for the analysis of platinum‐based chemotherapy did not provide sufficient power to arrive at statistically sound conclusions. Further studies are needed to explore the relationship between non‐BRCA HRR pathway mutations and the efficacy of chemotherapy and PARP inhibitor therapy.

## 5 CONCLUSIONS

Our data indicate that HRR pathway gene somatic mutations are common in Chinese patients with ovarian cancer. HRR pathway gene somatic mutations were associated with improved platinum‐based chemotherapy sensitivity. Large‐scale prospective studies are needed to verify our findings.

## AUTHOR CONTRIBUTIONS

**Zongbi Yi**: Conceptualization (equal); data curation (equal); formal analysis (equal); funding acquisition (equal); investigation (equal); methodology (equal); project administration (equal); resources (equal); software (equal); visualization (equal); writing – original draft (equal); writing – review and editing (equal). **Min Chen**: Data curation (equal); formal analysis (equal); writing – original draft (equal); writing – review and editing (equal). **Shaoxing Sun**: Data curation (equal); resources (equal); writing – review and editing (equal). **Chunxu Yang**: Data curation (equal); resources (equal); writing – review and editing (equal). **Zijie Mei**: Data curation (equal); resources (equal); writing – review and editing (equal). **Hui Yang**: Data curation (equal); formal analysis (equal); resources (equal); writing – review and editing (equal). **Qingming Xiang**: Conceptualization (equal); data curation (equal); formal analysis (equal); investigation (equal); methodology (equal); project administration (equal); resources (equal); software (equal); supervision (equal); validation (equal); visualization (equal); writing – original draft (equal); writing – review and editing (equal). **Hui Qiu**: Conceptualization (equal); data curation (equal); formal analysis (equal); investigation (equal); methodology (equal); project administration (equal); resources (equal); supervision (equal); validation (equal); visualization (equal); writing – original draft (equal); writing – review and editing (equal).

## CONFLICT OF INTEREST

The authors declare no conflicts of interest.

## ETHICS STATEMENT

The study protocol was reviewed and approved by the Institutional Review Board of the Department of Radiation and Medical Oncology, Zhongnan Hospital of Wuhan University (2020034).

## INFORMED CONSENT

All participants provided informed consent before participating in this study.

## Data Availability

The data used to support the findings of this study are available from the corresponding authors upon request.
